# A Feature-Encoded Physics-Informed Parameter Identification Neural Network for Musculoskeletal Systems

**DOI:** 10.1115/1.4055238

**Published:** 2022-09-19

**Authors:** Karan Taneja, Xiaolong He, QiZhi He, Xinlun Zhao, Yun-An Lin, Kenneth J. Loh, Jiun-Shyan Chen

**Affiliations:** Department of Structural Engineering, University of California San Diego, La Jolla, CA 92093; Department of Civil, Environmental, and Geo-Engineering, University of Minnesota, Minneapolis, MN 55455

**Keywords:** physics-informed neural networks, parameter identification, musculoskeletal system, data-driven computing, feature-encoding, surface electromyography

## Abstract

Identification of muscle-tendon force generation properties and muscle activities from physiological measurements, e.g., motion data and raw surface electromyography (sEMG), offers opportunities to construct a subject-specific musculoskeletal (MSK) digital twin system for health condition assessment and motion prediction. While machine learning approaches with capabilities in extracting complex features and patterns from a large amount of data have been applied to motion prediction given sEMG signals, the learned data-driven mapping is black-box and may not satisfy the underlying physics and has reduced generality. In this work, we propose a feature-encoded physics-informed parameter identification neural network (FEPI-PINN) for simultaneous prediction of motion and parameter identification of human MSK systems. In this approach, features of high-dimensional noisy sEMG signals are projected onto a low-dimensional noise-filtered embedding space for the enhancement of forwarding dynamics prediction. This FEPI-PINN model can be trained to relate sEMG signals to joint motion and simultaneously identify key MSK parameters. The numerical examples demonstrate that the proposed framework can effectively identify subject-specific muscle parameters and the trained physics-informed forward-dynamics surrogate yields accurate motion predictions of elbow flexion-extension motion that are in good agreement with the measured joint motion data.

## 1 Introduction

Human movement resulting from the interaction of various subsystems within the human body is controlled by the excitation signals from the central nervous system that lead to joint motion in the musculoskeletal (MSK) system. These subsystems, governed by parameterized nonlinear differential equations [1,2], form the forward dynamics problem [2]. Given information on muscle activations, the joint motion of a subject-specific MSK system can be obtained by solving a forward dynamics problem. The muscle activations can be estimated by the surface electromyography (sEMG) signals through a noninvasive procedure [2,3]. Surface electromyography driven forward dynamics have been widely used for predictions of joint kinetics or kinematics [2–5]. For rehabilitation applications in assessing muscular pathologies or weakened muscle groups, Pau et al. [4] used a simplified geometric model of the MSK system for elbow flexion to predict the motion of the elbow joint, given an sEMG signal. Zhao et al. [6] utilized sEMG signals to simulate wrist kinematics for various flexion/extension trials. Standard optimization techniques such as genetic algorithms [4,6], simulated annealing [7], and nonlinear least squares [2,3] have been used for parameter identification.

In recent years, machine learning (ML) or deep-learning-based approaches have become a viable alternative due to their flexibility and capability in extracting complex features and patterns from data [8] and have been successfully applied to various problems in engineering applications such as reduced-order modeling [9–13], and materials modeling [14,15], among others. For motion prediction, data-driven approaches have been introduced to directly map the input sEMG signal to joint kinetics/kinematics, bypassing the forward dynamics equations and the need for parameter estimation. For example, Au et al. [16] used only sEMG data as input to a time-delayed neural network (NN) to predict shoulder motion. Wu et al. [17] applied reinforcement learning for the sEMG and joint moment mapping. Leserri et al. [18] used features of the raw sEMG signals to map them to the elbow joint motion. Ma et al. and Ren et al. [19,20] used enhanced recurrent neural networks and convolutional neural networks, respectively, to map the raw sEMG signal to the motion of the upper limb. While these ML approaches do not require calibrating physiological parameters, the resulting ML-based surrogate models lack interpretability and may not satisfy the underlying physics.

To integrate data and physical models by using ML approaches, data-driven computing that enforces constraints of conservation laws in the learning algorithms of a material database has been developed in the field of computational mechanics [21–23]. This paradigm has been applied to other engineering problems, such as nonlinear material modeling [22,24,25] and, fracture mechanics [26], among others. Furthermore, deep manifold embedding techniques have been introduced in data-driven computing for extracting low-dimensional feature space [27,28].

More recently, physics-informed neural networks (PINNs) have been developed [29–32] to approximate the solutions of given physical equations by using feed-forward NNs and minimizing the residuals of the governing partial differential equations (PDEs) and the associated initial and boundary conditions. The PINN method has been successfully applied to problems such as flow and transport in porous media [31], solid mechanics [30], additive manufacturing [33], biomechanics, and biomedical applications [34–37], and inverse problems [29,30,38–40]. In inverse modeling with PINNs, the unknown system characteristics are considered trainable parameters or functions [29,41].

In this study, we propose a physics-informed parameter identification neural network (PI-PINN) for the simultaneous prediction of motion and parameter identification with application to MSK systems. Using the raw transient sEMG signals obtained from the sensors and the corresponding joint motion data, the PI-PINN learns to predict the motion and identifies the parameters of the hill-type muscle models representing the contractile muscle-tendon complex.

The standard PINNs with a fully connected architecture present difficulties in learning the high-frequency components of the solution, which is known as *spectral bias* [32,42,43]. Wong et al. [43] pointed out that PINNs tend to have a strong bias toward low-frequency patterns which are considered trivial solutions to the governing equations. To mitigate the issue of spectral bias, in this work a *Fourier feature* transformation is applied to modulate the signals input to embedding space [42,43]. A similar approach was proposed in Ref. [37] where a feature layer has been introduced for encoding certain dynamic patterns, such as periodicity. Inspired by these studies, we further propose a feature-encoded approach for the PI-PINN framework, termed feature-encoded physics-informed parameter identification neural network (FEPI-PINN), in dealing with the highly oscillatory sEMG signals. Here, the sEMG signals and joint motion data are first projected onto a low-dimensional space consisting of Fourier and polynomial bases. The NN is then used to map the associated basis coefficients between the input signal and the target output signal. Subsequently, the mapped coefficients are used to reconstruct the joint motion using the bases.

This paper is organized as follows. Section 2 introduces the subsystems and mathematical formulations of MSK forward dynamics, including the EMG-to-activation dynamics, the muscle-tendon contraction dynamics, and the MSK system dynamics, followed by an introduction of the proposed FEPI-PINN framework for simultaneous motion prediction and system parameter identification. Section 3 discusses the verification of the proposed algorithms using synthetic motion data. In Sec. 4, the proposed frameworks are validated by modeling the elbow flexion–extension movement using subject-specific sEMG signals and recorded motion data. Concluding remarks and future work are summarized in Sec. 5.

## 2 Methods

In this section, the mathematical formulations of subsystems for the forward dynamics of the human MSK system are described, followed by an introduction of the proposed PI-PINN framework designed for simultaneous forward dynamics learning and parameter identification.

### 2.1 Musculoskeletal Forward Dynamics.

**Fig. 1:**
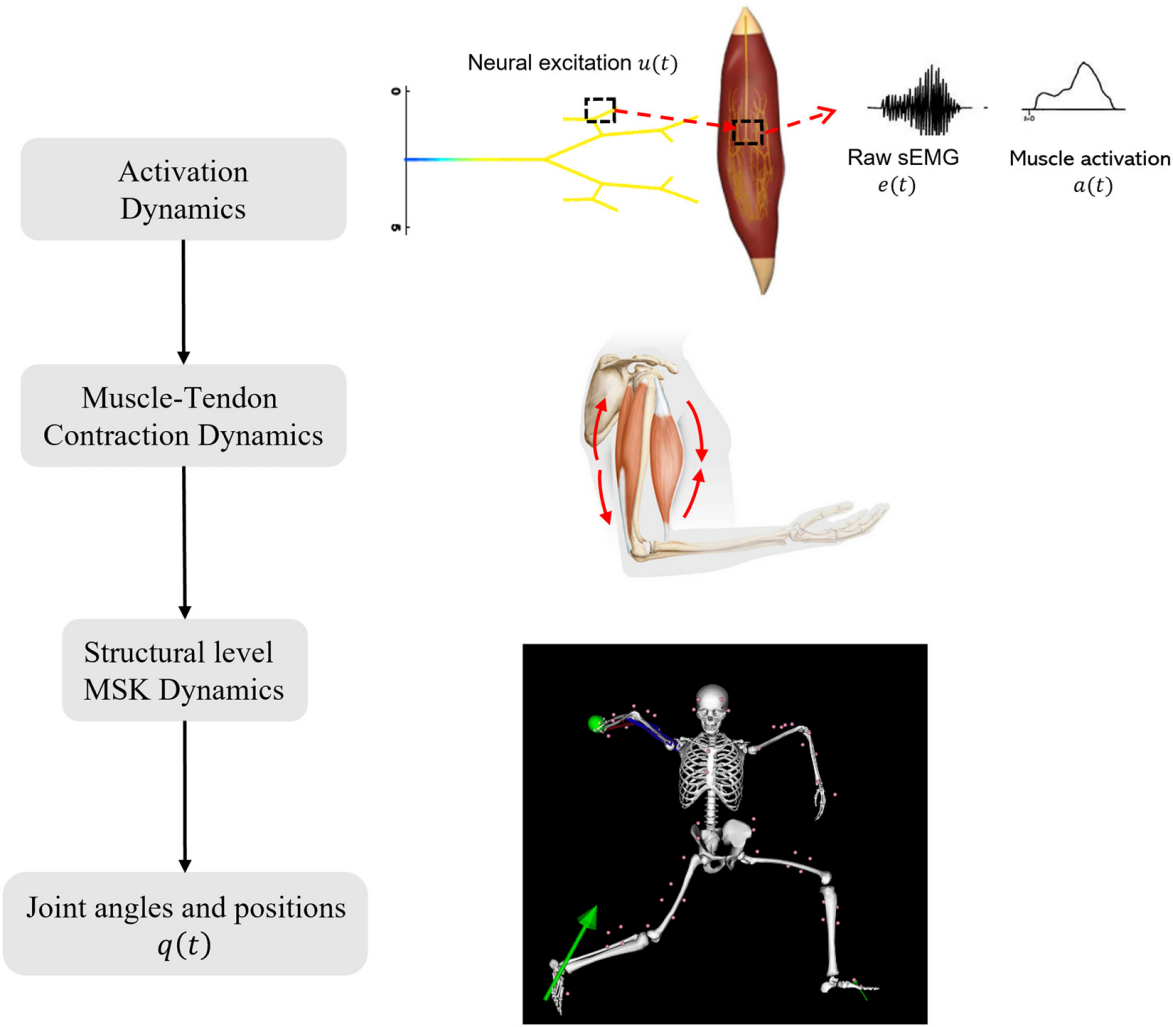
A flowchart depicting the interaction of the different subsystems related to the motion of the MSK system. Excitation from neurons is transmitted to muscle fibers (activation dynamics) that contract to produce force (muscle-tendon contraction dynamics). These forces generate torques at the joints (structural level MSK dynamics), leading to joint motion. The boxed entities are the nonlinear differential equations that relate to the different states of the system.

The hierarchical interaction of various subsystems of MSK forward dynamics is illustrated in Fig. 1, where the activation dynamics transform neural excitation (which can be measured by sEMG signals) to muscle activation that drives muscle fibers to produce force through the muscle-tendon (MT) contraction dynamics, leading to joint motion (translation and rotation) of MSK systems via the MSK system dynamics. The mathematical formulations of these subsystems are introduced in Secs. 2.1.1–2.1.3.

#### 2.1.1 EMG-to-Activation Dynamics.

The raw sEMG signals e(t) are measures of the neural excitation u(t) from the central nervous system. The EMG-to-Activation dynamics describes a nonlinear transformation from sEMG signals to muscle activations a(t) [4,6,44], which activate muscle fibers in a muscle group to produce active force. The neural excitation signal u(t) can be transformed from the sEMG signals e(t) by [2,3,45]

(1) u(t)=e(t−d)

where d is the electromechanical delay between the neural excitation originating and reaching the muscle group [45]. The muscle activation signal a(t) can then be obtained from the neural excitation u(t) in Eq. (1) by

(2) $$a(t)=\frac{\exp\left(Au(t)\right)-1}{\exp\left(A\right)-1}$$

where A is a shape factor [45].

#### 2.1.2 Muscle-Tendon Contraction Dynamics.

The MT contraction dynamics is introduced to relate the muscle contraction to force production via a hill-type muscle model [46,47] parameterized by the maximum isometric force in the muscle ( $f_{0}^{M}$), the optimal muscle length ( $l_{0}^{M}$) corresponding to the maximum isometric force, the maximum contraction velocity ( $v_{\max}^{M}$), the slack length of the tendon ( $l_{s}^{T})$, and the pennation angle ( ϕ) to be discussed as follows.

First, let each muscle group be characterized by a parameter vector

(3) $$\kappa =[l_{0}^{M},\;v_{\max}^{M},\;f_{0}^{M},\;l_{s}^{T},\;\phi ]$$

The force generated by the muscle group contains an active and a passive component [48,49]. The active force $f^{A}$ component can be expressed as

(4) $$\begin{array}{c} f^{A}(a,{\tilde{l}}^{M},{\tilde{v}}^{M};\kappa )=af^{A,L}({\tilde{l}}^{M};\kappa )f^{V}({\tilde{v}}^{M};\kappa )\\ {\tilde{l}}^{M}=l^{M}/l_{0}^{M}\\ {\tilde{v}}^{M}=v^{M}/v_{\max}^{M} \end{array}$$

where a is the activation function in Eq. (2), ${\tilde{l}}^{M}$ is the normalized muscle length, ${\tilde{v}}^{M}$ is the normalized velocity of the muscle, and $f^{A,L}({\tilde{l}}^{M})$ and $f^{V}({\tilde{v}}^{M})$ are functions of the length and velocity-dependent force generation properties of the active muscle represented by generic functions of dimensionless quantities as given in Appendix B [48–50], allowing them to be scaled to specific muscles through the parameters described. The total muscle force $F^{M}$ can be expressed as

(5) $$F^{M}(a,{\tilde{l}}^{M},{\tilde{v}}^{M};\kappa )=f_{0}^{M}(f^{A}(a,{\tilde{l}}^{M},{\tilde{v}}^{M};\kappa )+f^{P}({\tilde{l}}^{M};\kappa ))$$

where $f^{P}({\tilde{l}}^{M})$ is the passive muscle length-dependent force generation function with the specific form given in Appendix B.

**Fig. 2:**
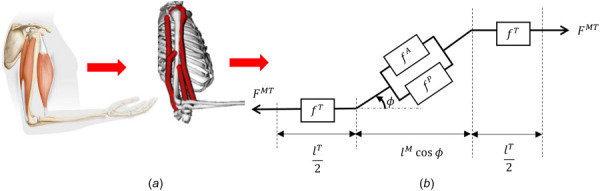
A representation of the muscle-tendon complex in the arm modeled by homogenized hill-type models. Each dark grey line in (*a*) is a homogenized MT complex described by the model shown in (*b*).

The muscle force $F^{M}$ obtained in Eq. (5) is transmitted to the joints through the tendon (Fig. 2(*b*)). The tendon produces force $F^{T}$ only when its length $l^{T}$ is stretched beyond its slack length $l_{s}^{T}$. In this study, the tendon is assumed to be rigid [6,47] and thus the tendon length $l^{T}=l_{s}^{T}$ is adopted. According to the schematics of the MT complex shown in Fig. 2(*b*), the total length of MT complex, $l^{\mathrm{MT}}$, is first obtained by

(6) $$l^{MT}=l_{s}^{T}+l^{M}\text{cos}\phi$$

where ϕ is the pennation angle. The total force produced by the MT complex, $F^{\mathrm{MT}}$, can be expressed as follows based on the force equilibrium:

(7) $$F^{\mathrm{MT}}(a,{\tilde{l}}^{M},{\tilde{v}}^{M},\phi ;\kappa )=F^{M}(a,{\tilde{l}}^{M},{\tilde{v}}^{M};\kappa )\text{cos}\phi$$

The rigid-tendon model simplifies the MT contraction dynamics [46,47,50] which accounts for the interaction of the activation, force-length, and force-velocity properties of the MT complex. More details on how muscle length and velocity are calculated can be found in Appendix A.

#### 2.1.3 Musculoskeletal System Dynamics.

The motion of the MSK system is modeled as the rotational movement of rigid links (bones) with force producing actuators (MT complex's). The force produced by these actuators is converted to torques at the joints of the body which ultimately leads to movement. This torque equilibrium can be expressed as

(8) $$I(q)\;\ddot{q}-T^{\mathrm{MT}}(a,q,\dot{q};\kappa )-E(q)=0$$

where $q,\;\dot{q},\;\ddot{q}$ are the vectors of generalized angular motions, angular velocities, and angular accelerations, respectively; E(q) is the torque from the external forces acting on the MSK system, e.g., ground reactions, gravitational loads, etc.; I(q) is the inertial matrix; $T^{MT}$ is the torque from all muscles in the model calculated by $T^{MT}(a,q,\dot{q};\kappa )=R(q)F^{MT}(a,q,\dot{q};\kappa )$, where R(q) are the moment arm's and $F^{MT}(a,q,\dot{q};\kappa )$ is obtained from Eq. (7). As the muscle lengths $l^{M}$ and velocities $v^{M}$ are functions of the joint motion q and $\dot{q}$, and the total force produced by all the MT complex's in the model is represented as $F^{\mathrm{MT}}(a,q,\dot{q};\kappa )$. Given the muscle activation signals from Eq. (2) and parameters of involved muscle groups, the generalized angular motions q and angular velocities $\dot{q}$ of the joints can be obtained by solving Eq. (8) [50].

### 2.2 Simultaneous Forward Dynamics Learning and Parameter Identification.

The proposed PI-PINN framework for simultaneous prediction of motion and parameter identification of human MSK systems is first introduced in Sec. 2.2.1, where the MSK forward dynamics are learned by an NN surrogate that predicts motion for identification of MSK properties using time-domain training. The enhanced forward dynamics surrogate is then introduced in Sec. 2.2.2, with the feature-encoded training.

With the governing equations for the MSK forward dynamics introduced in Sec. 2.1, the following parameterized ordinary differential equation (ODE) system is defined as

(9) L[q(t);λ]=b(t;ω) ∀ t∈ (0,T], B[q(0)]=g 

where the differential operator L[⋅;λ] is parameterized by a set of parameters $\lambda =\{\lambda_{0},\lambda_{1},\ldots ,\lambda_{n}\}$. The right-hand side b(t;ω) is parameterized by $\omega =[\omega_{1},\omega_{2},\ldots ,\omega_{m}]$. B[⋅] is the operator for initial conditions, and g is the vector of prescribed initial conditions. To simplify notations, the ODE parameters are denoted by Γ={λ,ω}. The solution to the ODE system q:[0,T]→R depends on the choice of parameters Γ.

#### 2.2.1 Forward Dynamics NN Surrogate With Time-Domain Training.

Here, a multilayer NN is used to relate data inputs containing discrete sEMG signals and discrete time, $x\in R^{n_{\mathrm{in}}}$, to discrete joint motion data outputs, $q\in R^{n_{\mathrm{out}}}$, by a nonlinear activation function h(·) as follows:

(10) $$a^{\left(k\right)}=h(W^{\left(k\right)}a^{\left(k-1\right)}+b^{\left(k\right)}),\;k=1,\ldots ,M$$

where M is the number of hidden layers; the superscript (k) denotes the layer number. Here, the activation $a^{\left(k\right)}\in R^{n_{k}}$ is the output of layer k with $n_{k}$ neurons. For the input layer, $n_{0}=n_{in}$ is the input dimension and $a^{\left(0\right)}=x$; $W^{\left(k\right)}\in R^{n_{k}\times n_{k-1}}$ and $b^{\left(k\right)}\in R^{n_{k}}$ are trainable weight and bias coefficients, respectively; θ={W,b} is used to denote all trainable parameters of the network, where $W={\left\{W^{\left(k\right)}\right\}}_{k=1}^{M+1}$ and $b={\left\{b^{\left(k\right)}\right\}}_{k=1}^{M+1}$ are the set of all weights and biases, respectively. A hyperbolic tangent activation function h(·) is used for the hidden layers, where the activation is applied to all components of its input vector. As the application of interest is a regression task, a linear activation is used for the output layer, which means the output of the last hidden layer is mapped to the prediction of the output layer

(11) $$\hat{q}=W^{\left(M+1\right)}a^{\left(M\right)}+b^{\left(M+1\right)}$$

where $\hat{q}$ is the approximation of the output training data q.

An NN approximates the MSK forward dynamics, which predicts MSK motion given the time history of raw sEMG signals of muscle groups. The raw sEMG signals inform the motion prediction with all transient information of sensor measured sEMG signals without any processing. Since we use the signals in their time-domain, we denote this as time-domain training.

Let us consider the $k^{\mathrm{th}}$ motion trial out of *p* trials of the training data of the MSK forward dynamics with $n_{k}$ data points or time-steps. For the surrogate, the input data of the $k^{\mathrm{th}}$ trial is $x_{k}={\left[x_{k}^{(1)T},\ldots ,\;x_{k}^{(n_{k})T}\right]}^{T}$ with the $i^{\mathrm{th}}$ row as $x_{k}^{(i)T}=[t_{k}^{\left(i\right)},e_{k,1}^{\left(i\right)},\ldots ,e_{k,N_{a}}^{\left(i\right)}]$ while the output data is $q_{k}={\left[q_{k}^{\left(1\right)},\;\ldots ,\;q_{k}^{\left(n_{k}\right)}\right]}^{T}$. Here, $t_{k}^{\left(i\right)}$ and $q_{k}^{\left(i\right)}$ denote the time and the MSK joint motion at the $i^{\mathrm{th}}$ time-step in the $k^{\mathrm{th}}$ trial, respectively, and ${\left\{e_{k,j}^{\left(i\right)}\right\}}_{j=1}^{N_{a}}$ denotes the set of the raw sEMG signals of $N_{a}$ muscle groups involved in the MSK joint motion $q_{k}^{\left(i\right)}$ at the $i^{th}$ time-step. The entire training dataset has $N_{\mathrm{data}}=\sum_{i=1}^{p}n_{i}$ data points with the input as $x={\left\lfloor x_{1},\;\ldots ,\;x_{p}\right\rfloor }^{T}$ and the target output as $q={\left\lfloor q_{1},\;\ldots ,\;q_{p}\right\rfloor }^{T}$.

**Fig. 3:**
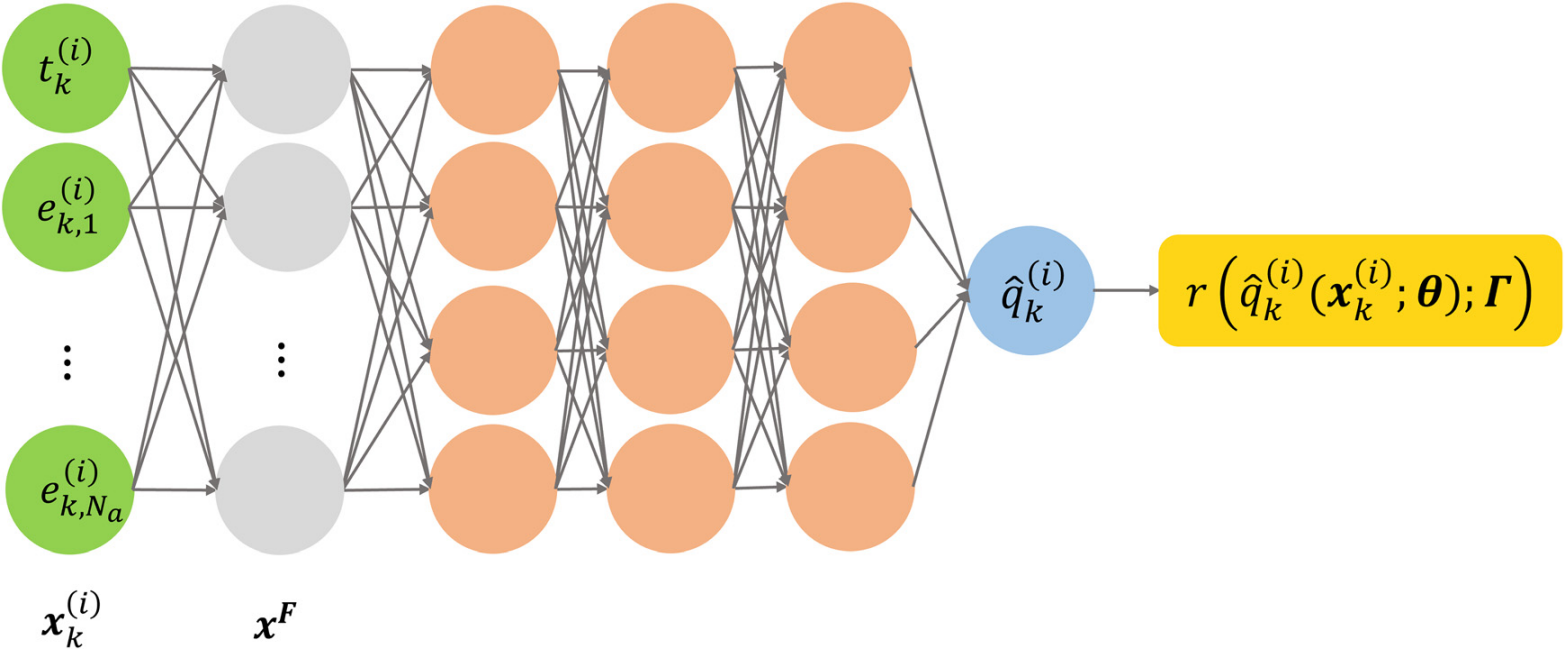
A computational graph of time-domain PI-PINNs with Fourier features prefixed to the network

The NN is trained to learn the mapping from the raw sEMG and time ( $x_{k}^{\left(i\right)}$) to the MSK joint motion ( $q_{k}^{\left(i\right)}$). The trainable parameters of the NN are obtained by minimizing the following loss function:

(12) $$J_{\mathrm{data}}=\frac{1}{N_{\mathrm{data}}}\sum_{i=1}^{p}{\left\|{\hat{q}}_{i}(x_{i};\theta )-q_{i}\right\|}_{L_{2}}^{2}$$

where ${\left\|\cdot \right\|}_{L_{2}}^{2}$ denotes the L2 norm. In addition to training an MSK forward dynamics surrogate, the proposed framework aims to simultaneously identify important MSK parameters from the training data by minimizing residual of the governing equation of MSK system dynamics in Eq. (8)

(13) $$J_{\mathrm{res}}=\frac{1}{N_{\mathrm{data}}}\sum_{i=1}^{p}{\left\|r({\hat{q}}_{i}(x_{i};\theta );\Gamma )\right\|}_{L_{2}}^{2}$$

where $r({\hat{q}}_{i}(x_{i};\theta );\Gamma )=L[{\hat{q}}_{i}(x_{i};\theta );\lambda ]-b(t^{\left(i\right)};\omega )$ denotes the vector of residuals associated with Eq. (8) for the $i^{\mathrm{th}}$ sample; ${\hat{q}}_{i}(x_{i};\theta )$ is the vector of predicted MSK joint motion from the NN with the trainable parameters θ for the $i^{\mathrm{th}}$ sample; Γ={λ,ω} represents the set of ODE parameters relevant to the MSK system. The optimal NN parameters $\tilde{\theta }$ and the ODE parameters $\tilde{\Gamma }$ are obtained by minimizing the composite loss function J as follows:

(14) $$\tilde{\theta },\tilde{\Gamma \;}=\underset{\theta ,\Gamma }{\mathrm{argmin}}\left(J\right)=\underset{\theta ,\Gamma }{\mathrm{argmin}}\left({\;J}_{\mathrm{data}}+{\beta \;J}_{\mathrm{res}}\right)$$

where β is the parameter to regularize the loss contribution from the ODE residual term in the loss function and is estimated analytically during the training [51]. For this study, β was scaled so that the terms $J_{\mathrm{res}}$ and ${\;J}_{\mathrm{data}}$ in the loss function given in Eq. (14) were with the same unit. The details are described in Sec. 3. The gradients of the NN outputs with respect to the NN MSK parameters (θ,Γ) can be obtained efficiently by automatic differentiation [52]. The computational graph is illustrated in Fig. 3.

##### 2.2.1.1 Fourier features.

It has been pointed out in the literature that training PINNs can be challenging as the solution could be trapped in a local minima, leading to large training data errors [42,43,51]. Wang et al. [42,51] showed that these networks tend to learn low-frequency components of the solution rather than the high-frequency counterparts, which is described as the spectral bias of PINNs. Wong et al. [43] further showed that PINNs are biased toward flat outputs which are considered as trivial solutions to the governing equations. It has been shown that employing Fourier features enables PINNs to learn the higher frequency components and avoid local minima, leading to improved learning performance [42]. PINNs with Fourier features have also been applied to image regression tasks in biomedical applications with high-dimensional data sources as magnetic resonance imaging [53]. Due to the oscillatory nature of the sEMG signals in the time-domain, the mapping to the MSK motion could contain features of various frequencies. Here, PINNs with Fourier features are employed in the forward dynamics surrogate with time-domain training.

A Fourier feature $x^{F}\in R^{m}$ of a given input $x\in R^{d}$ is defined as [42,43]

(15) $$x^{F}=\text{sin}\left(W^{F}x+b^{F}\right)$$

where $W^{F}\in R^{m\times d}$ and $b^{F}\in R^{m}$ are weight and bias coefficients, respectively, randomly sampled from a normal distribution $N(0,\sigma^{2})$, with σ as a tunable hyperparameter and sin(⋅) is the sinusoidal activation function. They can be easily added to the computational graph of PINN, as shown in Fig. 3. The equations for forward propagation remain the same with an additional Fourier feature transformation before the input layer. The optimization problem to obtain the optimal trainable network parameters $\tilde{\theta }$ and the ODE parameters $\tilde{\Gamma }$ remains the same as described in Eq. (14).

#### 2.2.2 Forward Dynamics NN Surrogate With Feature-Encoded Training.

Mapping oscillations in the input signal of the network to a smooth less-oscillatory output signal is susceptible to spectral bias. Given the high degree of oscillations in the transient raw sEMG signals, it was observed in the tests with time-domain training (Sec. 4) that the motion predictions were affected by artifacts of those oscillations. To this end, the framework was enhanced by encoding the features of the raw sEMG signals and motion signals to a low-dimensional space with Fourier and polynomial basis functions. This dimensionality reduction enables noise filtering and provides a smooth low-dimensional representation of the high-dimensional noisy data. A forward dynamics NN surrogate is then trained in the low-dimensional feature embedding space to perform mapping and simultaneously identify muscle parameters in the MSK system.

Due to the periodic nature of the signals, Fourier basis was used along with polynomials up to quadratic terms in the feature-encoded training. The polynomial basis helps capture the signals close to the boundary of the time-domain that have incomplete periodicity. For the $j^{\mathrm{th}}$ sEMG signal from the $k^{\mathrm{th}}$ trial, $e_{k,j}={\left\{e_{k,j}^{\left(i\right)}\right\}}_{i=1}^{n_{k}}$, consider the employment of $2n_{\mathrm{EMG}}$ Fourier basis terms with their respective coefficients $A_{k,j}={\left\{A_{k,j}^{\left(l\right)}\right\}}_{l=1}^{n_{EMG}}{\left\{B_{k,j}^{\left(l\right)}\right\}}_{l=1}^{n_{EMG}}$ and three coefficients for the complete quadratic basis terms, i.e., $C_{k,j}^{\left(0\right)},\;C_{k,j}^{\left(1\right)}$, and $C_{k,j}^{\left(2\right)}.$ The set of all input coefficients is denoted by $I_{k,j}$, defined as

(16) $$I_{k,j}=\{A_{k,j},B_{k,j},C_{k,j}^{\left(0\right)},C_{k,j}^{\left(1\right)},C_{k,j}^{\left(2\right)}\}$$

and the encoded approximation of the $j^{\mathrm{th}}$ sEMG signal from the $k^{\mathrm{th}}$ trial is given by

(17) $$e_{k,j}^{\mathrm{FE}}(t;I_{k,j})=\sum_{l=1}^{n_{\mathrm{EMG}}}\left[A_{k,j}^{\left(l\right)}\text{cos}\left(\frac{2\pi lt}{T_{k,j}^{e}}\right)+B_{k,j}^{\left(l\right)}\text{sin}\left(\frac{2\pi lt}{T_{k,j}^{e}}\right)\right]+C_{k,j}^{\left(0\right)}+C_{k,j}^{\left(1\right)}t+C_{k,j}^{\left(2\right)}t^{2}$$

where $T_{k,j}^{e}$ is the duration of the sEMG signal. Applying the encoding to all sEMG signals of the $k^{\mathrm{th}}$ trial gives a set of input coefficients, i.e., $I_{k}={\left\{I_{k,j}\right\}}_{j=1}^{N_{a}}$.

Correspondingly, there would be 2 $n_{q}+3$ coefficients for the encoded approximation of the $k^{\mathrm{th}}$ motion signal with the Fourier coefficients $F_{k}={\left\{F_{k}^{\left(l\right)}\right\}}_{l=1}^{n_{q}},\;G_{k}={\left\{G_{k}^{\left(l\right)}\right\}}_{l=1}^{n_{q}}$ and polynomial coefficients $H_{k}^{\left(0\right)},H_{k}^{\left(1\right)}$, and $H_{k}^{\left(2\right)}$. The set of coefficients $O_{k}$ is defined as

(18) $$O_{k}=\{F_{k},G_{k},H_{k}^{\left(0\right)},H_{k}^{\left(1\right)},H_{k}^{\left(2\right)}\}$$

(19) $$q_{k}^{\mathrm{FE}}(t;O_{k})=\sum_{l=1}^{n_{q}}\left[F_{k}^{\left(l\right)}\text{cos}\left(\frac{2\pi lt}{T_{k}^{q}}\right)+G_{k}^{\left(l\right)}\text{sin}\left(\frac{2\pi lt}{T_{k}^{q}}\right)\right]+H_{k}^{\left(0\right)}+H_{k}^{\left(1\right)}t+H_{k}^{\left(2\right)}t^{2}$$

where $T_{k}^{q}$ is the duration of the motion signal. The encoded coefficients of the approximations can be obtained by a least-squares minimization between the approximate and the original signals. An NN with *M* hidden layers is then trained to predict the output coefficients ${\hat{O}}_{k}$ by mapping the input coefficients $I_{k}$ to the target output coefficients $O_{k}$ as follows:

(20) $${\hat{O}}_{k}=W^{\left(M+1\right)}(h(W^{\left(M\right)}(\ldots h(W^{\left(1\right)}I_{k}+b^{\left(1\right)})\ldots )))+b^{\left(M+1\right)}$$

where $W={\left\{W^{\left(l\right)}\right\}}_{l=1}^{M+1}$, $\;b={\left\{b^{\left(k\right)}\right\}}_{l=1}^{M+1}$ and h(⋅) are the set of weights, set of biases, and activation functions (hyperbolic tangent) of the NN, respectively. Note that rather than learning the mapping related to transformed input features [37,42,43] in the time-domain, this feature-encoding approach is designed to learn the mapping of the basis coefficients, which can reduce mapping complexity and improve prediction accuracy as shown in our validation example.

**Fig. 4:**
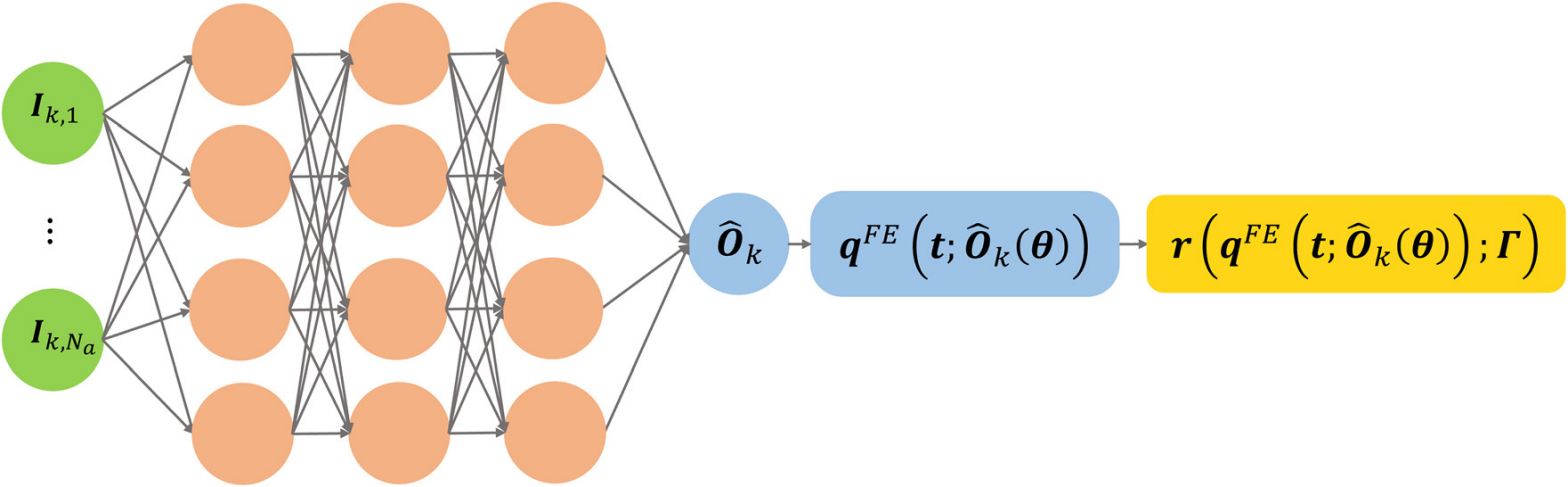
The computational graph for the FEPI-PINN framework

Given the predicted output coefficients $O_{k}$, the predictions of angular motion, velocity, and acceleration are obtained from Eq. (19) and its time derivatives. The loss function in Eq. (14) is modified as follows. The computational graph for the feature-encoded PI-PINN, termed FEPI-PINN, is shown in Fig. 4

(21) $$\tilde{\theta },\tilde{\Gamma \;}=\underset{\theta ,\Gamma }{\mathrm{argmin}}\left(J^{FE}\right)=\underset{\theta ,\Gamma }{\mathrm{argmin}}\left({\;J}_{\mathrm{data}}^{\mathrm{FE}}+\beta {\;J}_{\mathrm{res}}^{\mathrm{FE}}\right)$$

(22) $${\;J}_{\mathrm{data}}^{\mathrm{FE}}=\sum_{i=1}^{p}{\left\|\begin{array}{c} \begin{array}{c} {\hat{O}}_{i} \end{array}(I_{i};\theta ) \end{array}-O_{i}\right\|}_{L_{2}}^{2}$$

(23) $${\;J}_{\mathrm{res}}^{\mathrm{FE}}=\sum_{i=1}^{p}{\left\|\begin{array}{c} r(q^{FE}(t;\begin{array}{c} {\hat{O}}_{i} \end{array}(I_{i};\theta ));\Gamma ) \end{array}\right\|}_{L_{2}}^{2}$$

### 2.3 Sensor Data Acquisition.

**Fig. 5:**
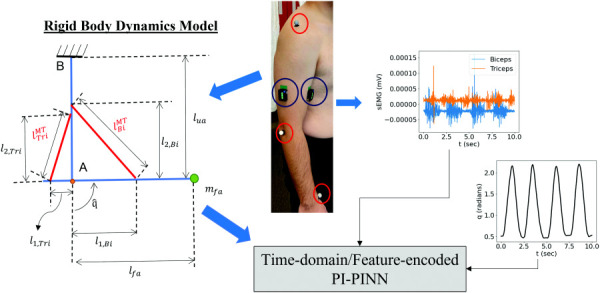
Overview of the application of this framework to the recorded motion data. The location of the motion capture markers is circled in red and the EMG sensors in blue. The simplified rigid body model is used in the forward dynamics equations within the framework with appropriately scaled anthropometric properties (for geometry) and physiological parameters (for muscle tendon material models). The raw sEMG signals are mapped to the target angular motion of the elbow and used to simultaneously characterize the MSK system using the proposed frameworks for PI-PINN.

The subject performs three trials of varying elbow flexion and extension motions as shown in Fig. 5, with each trial lasting 10 s. Three retroreflective markers with a diameter of 14 mm were attached to the acromion (shoulder), the humeral lateral epicondyle (elbow), and the radial styloid (wrist) of the right arm of the subject. Two delsys trigno surface sEMG sensors were affixed onto the biceps and triceps muscle groups, following surface electromyography for the non-invasive assessment of muscles project recommendations [54]. To minimize signal artifacts, the subject's arm was wiped with isopropyl alcohol before sEMG sensors were attached. The marker positions during the duration of the motion were captured by a Vicon motion capture system (Vicon Motion Systems Ltd., UK) at 150 Hz [55], and the sEMG signals were recorded at 2250 Hz. The kinematic data and the sEMG data were time-synchronized using the Vicon system via a trigger module. To obtain the muscle activations needed to calculate the MSK ODE residual, the raw sEMG signals were first centered with respect to their medians to center them around zero. The centered sEMG signals were processed using a Hampel filter to remove outliers resulting from skin artifacts, before going through a full-wave rectification. The rectified signals then go through a second-order Butterworth filter with a cutoff frequency of 3 Hz. Finally, the maximum voluntary contraction for each muscle was then taken as the maximum voltage from all trials for each muscle. The centered, rectified, and filtered sEMG signals were then normalized by the maximum voluntary contraction voltage to complete processing. They were then passed to the EMG-to-activation dynamics model for each muscle group to get the muscle activations as described in Sec. 2.1. The marker trajectories were directly used as is without any processing to estimate the elbow angle.

## 3 Verification: Elbow Flexion-Extension Motion

To verify the proposed PI-PINN framework, an elbow flexion-extension model was considered and the flowchart of the proposed PI-PINN computational framework for simultaneous forward dynamics prediction and parameter identification of MSK parameters is shown in Fig. 5. Synthetic sEMG signals and associated motion responses were considered and the time-domain PI-PINN approach was examined. The FEPI-PINN approach was employed in dealing with the recorded oscillatory sEMG data in the validation problem in Sec. 4.

The model contained two rigid links resembling the upper arm and forearm with lengths $l_{\mathrm{ua}}$ and $l_{\mathrm{fa}}$, respectively. Both rigid links were connected at a hinge resembling the elbow joint “A,” and the upper arm link was fixed at the top joint “B,” while the lines connecting the two links represented the biceps and triceps muscle-tendon complex, as shown in Fig. 2(*b*). The degree-of-freedom of the model was the elbow flexion angle q. The biceps and triceps muscle-tendon complex assemblies were modeled by two hill-type models with parameters $\kappa_{\mathrm{Bi}}$ and $\kappa_{\mathrm{Tri}}$, as discussed in Sec. 2.1. It was assumed that the mass in the forehand is concentrated at the wrist location, hence, a mass $m_{\mathrm{fa}}$ was attached to one end of the forearm link with a moment arm $l_{fa}$ from the elbow joint. We assumed that the tendons were rigid as discussed in Ref. [47] for ease of computation. The equation of motion for this rigid body system was

(24) $$I\ddot{q}=E(q)+T^{MT}(a_{\mathrm{Bi}},a_{\mathrm{Tri}},q,\dot{q};\kappa_{\mathrm{Bi}},\kappa_{\mathrm{Tri}})$$

where

(25) $$I=m_{fa}l_{fa}^{2}$$

(26) $$E(q)=-m_{\mathrm{fa}}gl_{\mathrm{fa}}\text{sin}\left(q\right)$$

(27) $$T^{MT}(a_{\mathrm{Bi}},a_{\mathrm{Tri}},q,\dot{q};\kappa_{\mathrm{Bi}},\kappa_{\mathrm{Tri}})=T_{\mathrm{Bi}}^{MT}(a_{\mathrm{Bi}},q,\dot{q};\kappa_{\mathrm{Bi}})-T_{\mathrm{Tri}}^{MT}(a_{\mathrm{Tri}},q,\dot{q};\kappa_{\mathrm{Tri}})$$

(28) $$T_{\mathrm{Bi}}^{MT}(a_{\mathrm{Bi}},q,\dot{q};\kappa_{\mathrm{Bi}})=\frac{F_{\mathrm{Bi}}^{MT}(a_{\mathrm{Bi}},{\tilde{l}}_{\mathrm{Bi}}^{M},{\tilde{v}}_{\mathrm{Bi}}^{M},{\tilde{l}}_{\mathrm{Bi}}^{T};\kappa_{\mathrm{Bi}})l_{2,\mathrm{Bi}}\text{sin}\left(q\right)l_{1,\mathrm{Bi}}}{l_{\mathrm{Bi}}^{MT}(q)}$$

(29) $$T_{\mathrm{Tri}}^{MT}(a_{\mathrm{Tri}},q,\dot{q};\kappa_{\mathrm{Tri}})=\frac{F_{\mathrm{Tri}}^{MT}(a_{\mathrm{Tri}},{\tilde{l}}_{\mathrm{Tri}}^{M},{\tilde{v}}_{\mathrm{Tri}}^{M},{\tilde{l}}_{\mathrm{Tri}}^{T};\kappa_{\mathrm{Tri}})l_{2,\mathrm{Tri}}\text{sin}\left(q\right)l_{1,\mathrm{Tri}}}{l_{\mathrm{Tri}}^{MT}(q)}$$

with the initial conditions $q(0)=\frac{\pi }{6}\;\mathrm{radians}$ and $\dot{q}(0)=0\;\mathrm{radians}/\sec$. Given the synthetic sEMG signals ( $e_{\mathrm{Bi}},e_{\mathrm{Tri}}$) that were plugged into the sEMG-to-activation dynamics equations (Sec. 2.1.1), leading to muscle activations $a_{\mathrm{Bi}},\;a_{\mathrm{tri}}$ and using the parameters summarized in Table 1, the motion of the elbow joint, q, was obtained by solving the MSK forward dynamics problem using a synthetic solver.

**Table 1:** Parameters involved in the forward dynamics setup of elbow flexion-extension motion

Parameter	Type	Value	Parameter	Type	Value
$l_{0,\mathrm{Bi}}^{M}$	Biceps muscle model	0.6 m	$m_{\mathrm{fa}}$	Equation of motion	1.0 kg
$v_{\max,\mathrm{Bi}}^{M}$	Biceps muscle model	6 m /sec	$l_{\mathrm{ua}}$	Geometric	1.0 m
$f_{0,\mathrm{Bi}}^{M}$	Biceps muscle model	300 N	$l_{\mathrm{fa}}$	Geometric	1.0 m
$l_{s,\mathrm{Bi}}^{T}$	Biceps muscle model	0.55 m	$l_{1,\mathrm{Bi}}$	Geometric	0.3 m
$\phi_{\mathrm{Bi}}$	Biceps muscle model	0.0 radians	$l_{2,\mathrm{Bi}}$	Geometric	0.8 m
$l_{0,\mathrm{Tri}}^{M}$	Triceps muscle model	0.4 m	$l_{1,\mathrm{Tri}}$	Geometric	0.2 m
$v_{\max,\mathrm{Tri}}^{M}$	Triceps muscle model	4 m /sec	$l_{2,\mathrm{Tri}}$	Geometric	0.7 m
$f_{0,\mathrm{Tri}}^{M}$	Triceps muscle model	300 N	d	Activation dynamics	0.08 sec
$l_{s,\mathrm{Tri}}^{T}$	Triceps muscle model	0.33 m	A	Activation dynamics	0.2
$\phi_{\mathrm{Tri}}$	Triceps muscle model	0.0 radians			

**Fig. 6:**
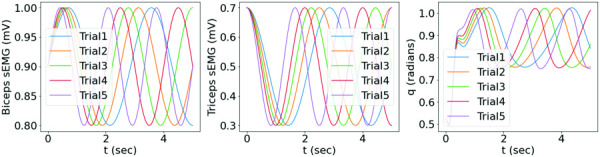
The dataset with synthetic bicep and tricep sEMG signals with variations in frequency for all trials, and the corresponding motion from the two

Five synthetic samples of the elbow flexion-extension motion were generated by solving Eq. (24) given synthetic muscle sEMG signals, as shown in Fig. 6. The training dataset contained the data of trials 1, 2, 4, and 5, while the data of trial 3 was used for testing, each trial with n=500 data points. The data of the $k^{\mathrm{th}}$ sample with n temporal data points contained $[x_{k},q_{k}]=[t_{k},e_{k,\mathrm{Bi}},e_{k,\mathrm{Tri}},q_{k}]\equiv {\left\{t_{k}^{\left(i\right)},e_{k,\mathrm{Bi}}^{\left(i\right)},e_{k,\mathrm{Tri}}^{\left(i\right)},q_{k}^{\left(i\right)}\right\}}_{i=1}^{n}$

The set of MSK parameters $\Gamma ={\left\{\Gamma_{l}\right\}}_{l=1}^{4}=\{f_{0,\mathrm{Bi}}^{M},v_{\max,\mathrm{Bi}}^{M},f_{0,\mathrm{Tri}}^{M},v_{\max,\mathrm{Tri}}^{M}\}$ were considered to be identified from the training data using the proposed framework. Due to different units and physiological nature of the parameters, they could be very different in terms of scale. For example, there could be a difference of more than two orders of magnitude between $f_{0}^{M}$ and $v_{\max}^{M}$, which could affect the conditioning of the parameter identification system. To mitigate this issue, normalization [40] was applied to each of the parameters

(30) $${\bar{\Gamma }}_{l}=\frac{\Gamma_{l}}{\Gamma_{l}^{\left(0\right)}}$$

where $\Gamma_{l}^{\left(0\right)}$ was the initial value of the parameter. Therefore, the set of parameters to be identified became $\bar{\Gamma }={\left\{\Gamma_{l}\right\}}_{l=1}^{4}$.

The proposed PI-PINN framework, as described in Sec. 2.2, was applied to simultaneously learn the MSK forward dynamics surrogate and identify the MSK parameters $\bar{\Gamma }$ by optimizing Eq. (14), where the residual of the governing equation for the training input $x_{k}^{\left(i\right)}$, was expressed as

r(q^k(xk(i);θq),q^˙k(xk(i);θq),q^¨k(xk(i);θq);Γ(Γ;Γ(0)))

(31) $$=I{\ddot{\hat{q}}}_{k}(x_{k}^{\left(i\right)};\theta_{q})-E({\hat{q}}_{k}(x_{k}^{\left(i\right)};\theta_{q}))-T^{MT}(a_{\mathrm{Bi}}(t^{\left(i\right)}),a_{\mathrm{Tri}}(t^{\left(i\right)}),{\hat{q}}_{k}(x_{k}^{\left(i\right)};\theta_{q}),{\dot{\hat{q}}}_{k}(x_{k}^{\left(i\right)};\theta_{q});\Gamma (\bar{\Gamma };\Gamma^{\left(0\right)}))$$

and could be plugged into the residual term $J_{\mathrm{res}}$ in the loss function in Eq. (14).

The residual of the equation of motion $\left({\;J}_{\mathrm{res}}\right)$ involved in the loss function J (Eq. (14)) was scaled so that the terms $J_{\mathrm{data}}$ and ${\;\beta J}_{\mathrm{res}}$ in Eq. (14) had the same unit. This yielded the scaling parameter $\beta \propto \frac{\Delta t^{2}}{I}$, where Δt is the time-step size in the time-signal and I is the moment of inertia of the mass around the elbow.

**Fig. 7:**
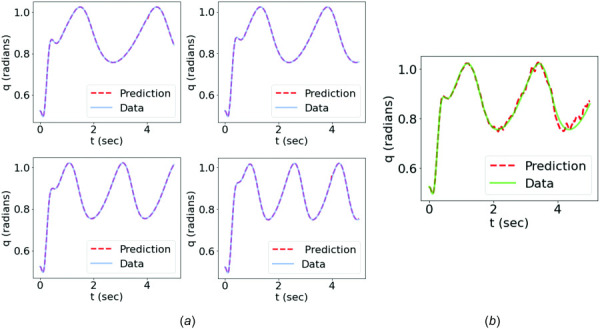
Comparison between the data of joint kinematics and the predictions from the trained forward dynamics surrogate: (*a*) the training cases and (*b*) the testing case

**Fig. 8:**
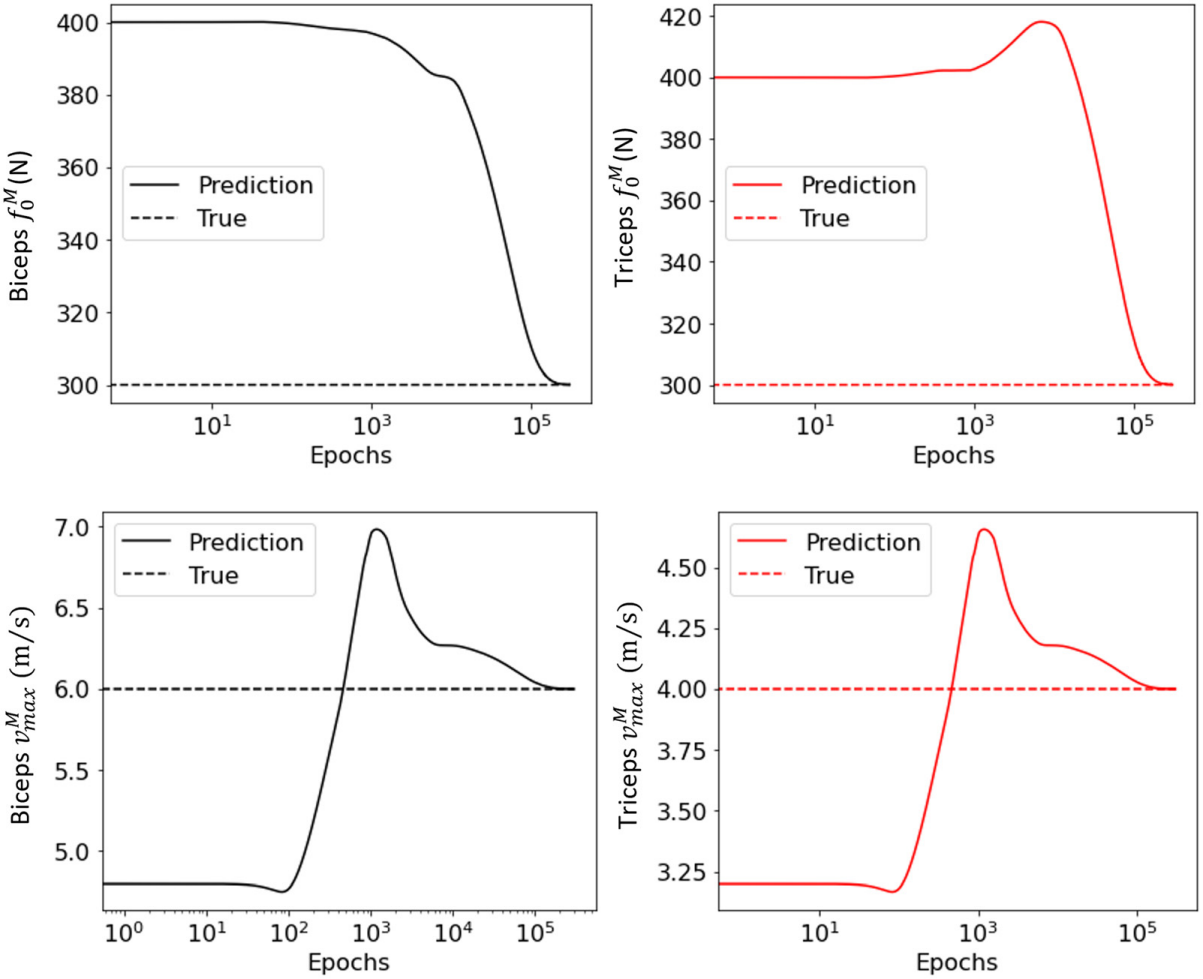
Evolution of the MSK parameters, $f_{0}^{M}$ and $v_{\max}^{M}$, of bicep and triceps during the training process

A three-layer feedforward NN with 32 neurons in each layer was used. The training was performed using the Adam algorithm [56] with an initial learning rate of $5{\times 10}^{-3}$. In this example, $\beta \propto \frac{\Delta t^{2}}{I}=10^{-4}$. The trained forward dynamics surrogate could accurately predict the joint kinematics given an input signal from the testing data, as shown in Fig. 7(*b*). Meanwhile, the MSK parameters, $f_{0}^{M}$ and $v_{\max}^{M}$, of both the biceps and the triceps were accurately identified from the motion data, with an error of less than 1%, as shown in Fig. 8. A sensitivity study of the results of this verification problem with respect to *β* is shown in Appendix C, where the study showed that this estimate of $\beta \propto \frac{\Delta t^{2}}{I}=10^{-4}$ lies within a range leading to optimal motion prediction and parameter identification from the framework.

## 4 Validation: Elbow Flexion–Extension Motion

**Fig. 9:**
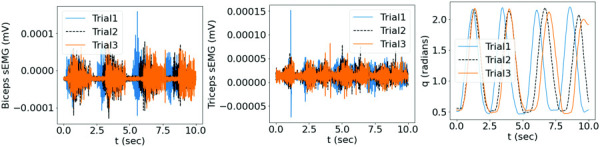
The measured transient raw sEMG signals and the corresponding angular motion of the elbow flexion-extension of the subject

The recorded motion data and sEMG signals were collected as per the data acquisition protocols mentioned in Sec. 2.3. The subject performed three trials of the elbow flexion-extension motion, where the sEMG sensors were fixed on two muscle groups, namely, the biceps and triceps. The sEMG signals were processed as described in Sec. 2.1 to obtain muscle activation signals for each muscle group that was used to calculate the MSK ODE residual. We used the same simplified rigid body model as in Sec. 3 and appropriately scaled the anthropometric properties (for the geometry of the model) and physiological parameters (for muscle-tendon material models used for the muscle groups) based on the generic upper body model defined in Refs. [50] and [57]. Figure 9 shows the measured data of the three trials, including the transient raw sEMG signals and the corresponding angular motion of the elbow flexion-extension of the subject.

In this example, the raw sEMG signals were used as input. The time-domain and the feature-encoded PI-PINN frameworks were examined and compared. The data of trials 1 and 3 were used for training, while trial 2 was used for validation, where each signal contained 1000 temporal data points. The normalization described in Eqs. (30) and (31) were adopted. The set of muscle parameters to be identified by the framework include the maximum isometric force and the maximum contraction velocity from both muscle groups, which are denoted as $\;\Gamma =\{f_{0,\mathrm{Bi}}^{M},v_{\max,\mathrm{Bi}}^{M},f_{0,\mathrm{Tri}}^{M},v_{\max,\mathrm{Tri}}^{M}\}$. The training was performed using the Adam algorithm [56] with an initial learning rate of $5{\times 10}^{-3}$.

### 4.1 Time-Domain PI-PINN Approach.

For the time-domain training, the training data of the $k^{\mathrm{th}}$ sample with $n_{k}$ data points contained $\left[x_{k},q_{k}]=[t_{k},e_{k,\mathrm{Bi}},e_{k,\mathrm{Tri}},q_{k}\right]$. The entire training dataset with a total of $N_{\mathrm{data}}=n_{1}+n_{3}$ data points could then be defined as follows: the input discrete time and sEMG signals were $t={\left[t_{1}^{T},t_{3}^{T}\right]}^{T}$, $e_{Bi}={\left[e_{1,\mathrm{Bi}}^{T},e_{3,\mathrm{Bi}}^{T}\right]}^{T}$, and $e_{\mathrm{Tri}}={\left[e_{1,\mathrm{Tri}}^{T},e_{3,\mathrm{Tri}}^{T}\right]}^{T}$. The output was the corresponding angular motion data $q={\left[q_{1}^{T},q_{3}^{T}\right]}^{T}$. An NN with three hidden layers, with 64 neurons in each layer, was used. The Fourier features used in the time-domain training were controlled by parameter σ=1.

**Fig. 10:**
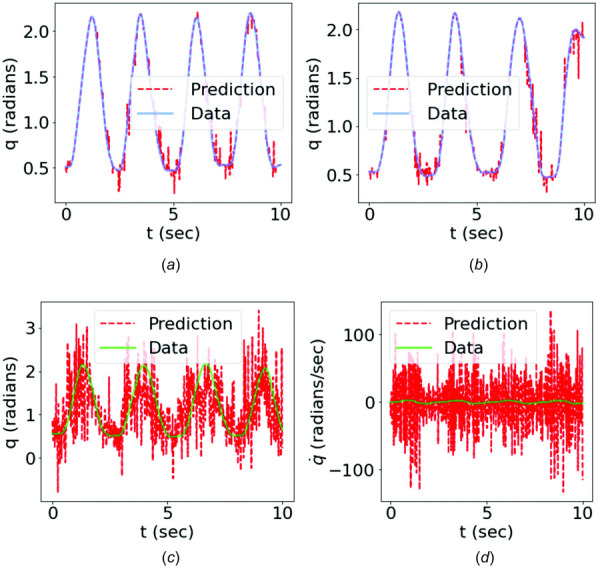
Comparison of the angular motion data with the predictions from the time-domain PI-PINN: (*a*) training case: trial 1; (*b*) training case: trial 3; (*c*) testing case: trial 2. The comparison of angular velocity of the testing case is shown in (*d*).

Figure 10 compares the data of joint kinematics with the predictions from the trained forward dynamics surrogate with time-domain training on both training and testing cases. The noise and oscillations in the raw sEMG signals pose difficulties for the NN surrogate in learning accurate mappings, leading to oscillatory and nonphysiological motion predictions, especially in the testing case. Nevertheless, the motion prediction captured the essential motion pattern, with the mean predicted motion trajectory close to that of the data.

### 4.2 Feature-Encoded PI-PINN (FEPI-PINN) Approach.

For the feature-encoded approach, the set of training data of the $k^{\mathrm{th}}$ sample contained $\left\{I_{k,\mathrm{Bi}},I_{k,\mathrm{Tri}},O_{k}\right\}$. The input coefficients of the training data sEMG signals of the biceps and triceps muscles were $I_{\mathrm{Bi}}={\left[I_{1,\mathrm{Bi}}^{T},I_{3,\mathrm{Bi}}^{T}\right]}^{T},\;\mathrm{and}\;I_{\mathrm{Tri}}={\left[I_{1,Bi}^{T},I_{3,Tri}^{T}\right]}^{T}$. The corresponding output coefficients were $O={\left[O_{1}^{T},O_{3}^{T}\right]}^{T}$. The number of Fourier basis terms used for the feature encoding of sEMG signals were $n_{\mathrm{EMG}}=1000$ while for the motion, $n_{q}=100$ were used. An NN with two hidden layers, 32 neurons in each layer, was employed.

**Fig. 11:**
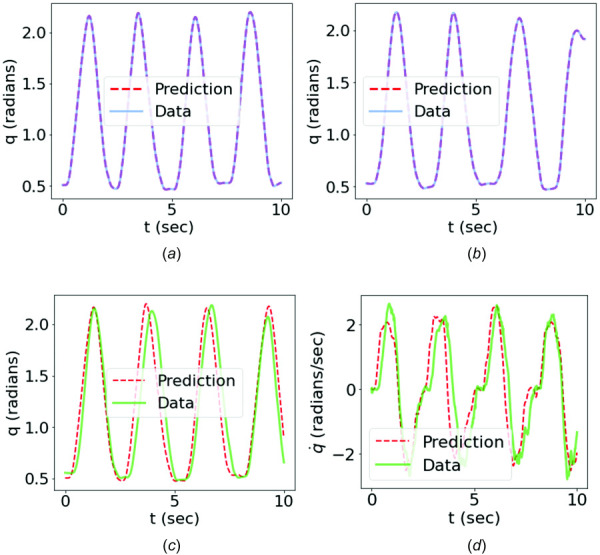
Comparison of the angular motion data with the predictions from the FEPI-PINN: (*a*) training case: trial 1; (*b*) training case: trial 3; (*c*) testing case: trial 2. The comparison of angular velocity of the testing case is shown in (*d*).

Figure 11 shows that a good agreement between the predictions and motion data was achieved for both training and testing cases using the feature encoding method. It demonstrated that the trained forward dynamics surrogate could learn the MSK forward dynamics and effectively predict the joint kinematics given a sEMG signal of the same type of motion used for training. Compared with the results obtained from the time-domain training, as shown in Fig. 10, the feature-encoded training showed a significant improvement in terms of prediction accuracy, which was attributed to the dimensionality reduction that filters out unnecessary noise and oscillations of original high-dimensional data. Figures 10(*c*) and 10(*d*) and Figs. 11(*c*), and 11(*d*) compare the data with the predictions of both methods in terms of angular motion q and velocity $\dot{q}$. Using exact derivatives in the feature-encoded training also led to smoother $\dot{q}$ values as compared to the ones obtained through the NN in time-domain training. It is noted that the motion prediction on the testing case was with a slight error, which could be attributed to the small size of the training dataset (i.e., using two trials). Overall, the feature-encoded training performs better in terms of motion prediction as shown in these figures and Table 2 where the error statistics are reported. The testing predictions from both training methods were compared to the true data and lower mean squared error and $R^{2}$ statistic close to 1 were seen in the case of the feature encoding. Here, the mean squared error (MSE) and $R^{2}$ statistic are defined as

(32) $$\mathrm{MSE}=\frac{1}{n_{2}}{\left\|\begin{array}{c} q-\hat{q} \end{array}\right\|}_{L_{2}}^{2}$$

(33) $$R^{2}=1-\frac{\sum_{i=1}^{n_{2}}{\left(q^{\left(i\right)}-{\hat{q}}^{\left(i\right)}\right)}^{2}}{\sum_{i=1}^{n_{2}}{\left(q^{\left(i\right)}-\bar{q}\right)}^{2}}$$

where $q={\left[q^{\left(1\right)},\ldots ,q^{\left(n_{2}\right)}\right]}^{T}$ is the motion data of trial 2, $\hat{q}={\left[{\hat{q}}^{\left(1\right)},\ldots ,{\hat{q}}^{\left(n_{2}\right)}\right]}^{T}$ is the prediction from the PI-PINN framework, and $\bar{q}$ is the mean of trial 2 s motion data.

**Table 2:** Statistics comparing the testing predictions of the framework with original time-domain training versus the enhanced feature-encoded training

Kinematics	Framework	MSE	$R^{2}$ score
Angular motion	Time-domain	0.50	–0.45
	Feature-encoded	0.03	0.91
Angular velocity	Time-domain	1973.75	–826.95
	Feature-encoded	0.37	0.84

### 4.3 Parameter Identification.

**Fig. 12:**
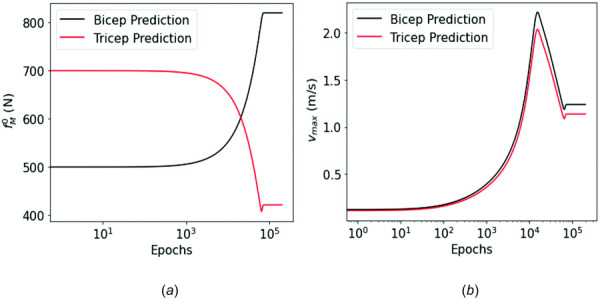
Evolution of the identified MSK parameters: (*a*) $f_{0}^{M}$ and (*b*) $v_{\max}^{M}$ of bicep and triceps during the training process. It shows the identified parameters converge to estimates that are in the physiological range of those parameters, as summarized in Table 3.

The identified MSK parameters from the FEPI-PINN that yielded higher accuracy of motion predictions are summarized in Table 3. Figure 12 shows the evolution of the MSK parameters, $f_{0}^{M}$ and $v_{\max}^{M}$, of both the biceps and the triceps during optimization, with the final converged values of $f_{0}^{M}$ lying within the physiological estimates of these parameters reported in the literature [57–59]. Note that since it is difficult to measure the parameter $v_{\max}^{M}$ in experiments, an estimate of $v_{\max}^{M}$, $8-10\;l_{0}^{M}/\sec,$ was typically used in hill-type muscle models [50]. Given that $l_{0,\mathrm{Bi}}^{M}=0.1157\;m$ and $l_{0,\mathrm{Tri}}^{M}=0.1138\;m$ were used in this model, the physiologically reasonable estimated range of $v_{\max,\mathrm{Bi}}^{M}$ and $v_{\max,\mathrm{Tri}}^{M}$ were 1.16–1.32 m/s and 1.14–1.34 m/s, respectively. The $v_{\max,\mathrm{Bi}}^{M}$ and $v_{\max,\mathrm{Tri}}^{M}$ identified by the proposed framework were 1.24 m/s and 1.138 m/s, respectively, as summarized in Table 3, which shows good agreement. The results demonstrated the effectiveness of the proposed FEPI-PINN framework and promising potential for real applications.

**Table 3:** The identified parameter estimates using feature encoding training, and their values reported in the literature [57–59]

Parameter	Identified values	Estimates from literature
$f_{0,\mathrm{Bi}}^{M}\;(N)$	819.56	525–849.29
$v_{\max,\mathrm{Bi}}^{M}\;(m/s)$	1.24	1.16–1.32
$f_{0,\mathrm{Tri}}^{M}\;(N)$	421.37	504–1176
$v_{\max,\mathrm{Tri}}^{M}\;(m/s)$	1.138	1.14–1.34

## 5 Discussion and Conclusions

In this work, a physics-informed parameter identification neural network (PI-PINN) framework, as well as its feature-encoded version (FEPI-PINN), was proposed for motion prediction and parameter identification of MSK systems. In this approach, the recorded marker data for positional information and raw signals from the sEMG sensors (for estimation of the activity of the two muscle groups involved in the motion) were utilized for training. The framework was built using a neural network that learns the mapping between the raw sEMG signals and joint kinematics, minimizing a loss function that consists of the error in the training data and the residual of the MSK forward dynamics equilibrium. Under this PI-PINN framework, time-domain training was first investigated. In this approach, sEMG and motion signals were mapped for their entire time duration, and Fourier features were used to learn the relationship between them as a forward dynamics surrogate. Next, an alternative FEPI-PINN approach was proposed to enhance the model performance by introducing a feature transformation encoder to project the noisy and oscillatory raw data onto a low-dimensional feature embedding space. In this feature-encoded PI-PINN (i.e., FEPI-PINN) framework, the forward dynamics surrogate relates basis coefficients of inputs to those of target outputs encoded in noise-filtered embedding space, yielding an enhanced prediction accuracy.

The time-domain method was first verified with synthetic data where PI-PINN was trained to learn the motion of elbow flexion-extension given synthetic sEMG signals. Simultaneously, the parameters related to the maximum isometric force and maximum contraction velocity in the hill-type models of the biceps and triceps muscle groups were also identified. In the validation problem, the elbow flexion-extension model was scaled according to the subject's anthropometric geometry. The recorded raw sEMG signal was employed as input to predict the angular motion. It was found that the time-domain PI-PINN training led to nonphysiological motion predictions due to the noise and oscillations in the raw sEMG signal that increases mapping complexities. FEPI-PINN, on the other hand, yielded smoother and physiologically accurate mappings of the angular motion and angular velocity. It was noticeable that, despite the limited size of the dataset, the FEPI-PINN approach outperformed the time-domain PI-PINN approach. A reasonable agreement was found between the identified parameters and the range of their physiological parameter estimates.

The applications of this technique are aimed at assistive devices or exoskeletons [20] that rely on guiding the motion of the subject based on control signals such as sEMG that inform muscle activity. Accurate prediction of the intended motion of the subject, based on their muscle activity is crucial for the successful implementation of these technologies. While in the earlier studies, methods have been proposed to map the sEMG signals to the joint motion by using deep learning techniques such as convolutional neural networks, recurrent neural networks, and auto-encoders [19,60,61], these black-box ML-based models do not satisfy the underlying physics and often lack interpretability. The proposed FEPI-PINN framework, on the other hand, provides a systematic approach to enforce the underlying physics into the mapping, improving the interpretability of ML-based motion. This study also suggested the potential extension of the FEPI-PINN framework for MSK digital twin applications. Under this framework, upon completion of model training with sufficient data from the subject(s), the characterized MSK and NN parameters provide a real-time model for joint motion prediction using sEMG signals without solving the nonlinear ODEs, as long as the sEMG signals are within the range of the training data. The characterized MSK physiology parameters also offer the potential diagnosis for muscle injury and disease development.

While a simplified MSK model was used to model the elbow flexion-extension movement, a more physiologically accurate representation of muscle tissues with fats, connective tissues, and muscle fibers could be used to inform the length and velocity-dependent force generation capacity of the muscles [62] in future work. To incorporate different types of motions and account for subject variability, transfer learning [63] could be applied to carry over the features from the previously learned motions for model enhancement.

## Figures and Tables

**Fig. 13:**
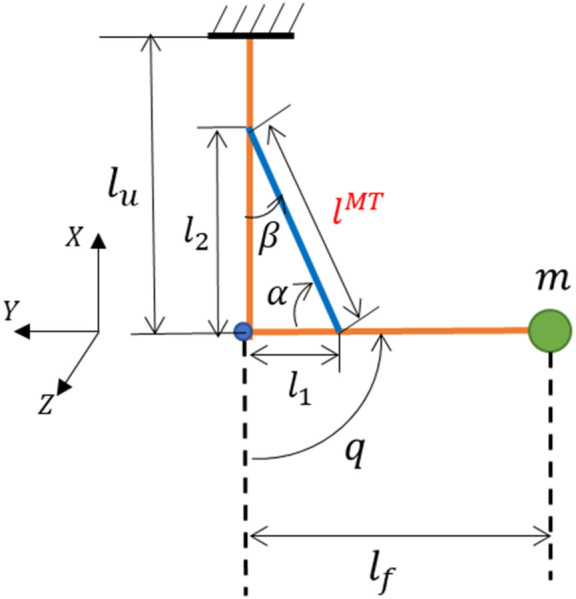
Simplified model to explain geometrical relations between $l^{M}$ and $l^{\mathrm{MT}}$

**Fig. 14:**
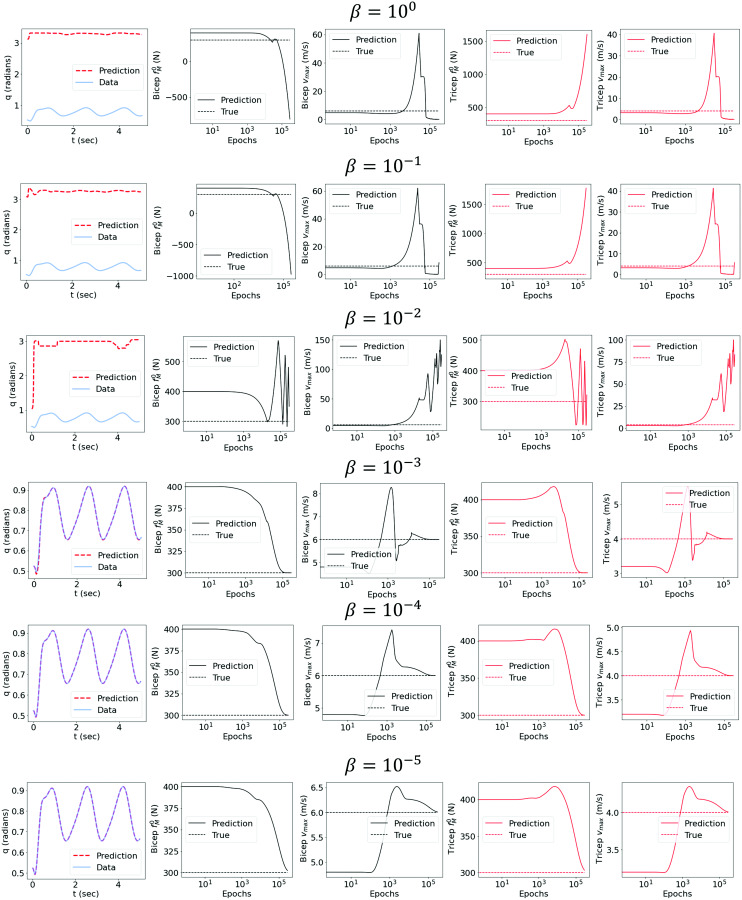
Results of the sensitivity study showing the range of β within which the framework shows stable results

## Nomenclature

a =: muscle activations
A =: shape factor used to relate neural excitation to muscle activation
d =: electromechanical delay between origin of neural excitation from central nervous system and reaching the muscle group
e =: raw sEMG signals captured by sensors
$f^{A}$ =: active force component of hill-type muscle model
$f^{A,L}$ =: length-dependent active force generation component
$f^{P}\;$ =: passive force component of hill-type muscle model
$f^{V}$ =: velocity-dependent active force generation component
$f_{0}^{M}$ =: maximum isometric force in the muscle
$F^{M}$ =: total muscle force
$F^{\mathrm{MT}}$ =: total force produced by muscle-tendon complex
$I_{k,j}$ =: set of all input feature-encoded basis coefficients of the $j^{\mathrm{th}}$ sEMG signal from the $k^{\mathrm{th}}$ trial
J =: composite loss function of the PI-PINN minimizing data and ODE residual
${\tilde{l}}^{M}$ =: normalized muscle length
$l^{\mathrm{MT}}$ =: total length of muscle-tendon complex
$l_{0}^{M}$ =: optimal muscle length corresponding to the maximum isometric force
$l_{s}^{T}$ =: slack length of the tendon
$l_{\mathrm{fa}}$ =: forearm length
$l_{\mathrm{ua}}$ =: upper arm length
$m_{\mathrm{fa}}$ =: mass of forearm
$O_{k}$ =: set of all output feature-encoded basis coefficients of the $k^{\mathrm{th}}$ trial
${\hat{O}}_{k}$ =: predicted output feature-encoded basis coefficients of the $k^{\mathrm{th}}$ trial
q =: generalized angular motion of the MSK system
${\hat{q}}_{k}$ =: predicted angular motion of the $k^{\mathrm{th}}$ trial from the PI-PINN framework
$T^{\mathrm{MT}}$ =: torque produced by the muscle-tendon complex
${\tilde{v}}^{M}$ =: normalized muscle velocity
$v_{\max}^{M}$ =: maximum contraction velocity
u =: neural excitations
$x_{k}$ =: input data from the $k^{\mathrm{th}}$ motion trial
$x^{F}$ =: Fourier feature vector
$\Gamma_{i}$ =: $i^{\mathrm{th}}$ parameter characterizing the parameterized ODE system
θ =: set of weights and biases of the neural network
κ =: vector of muscle parameters for each muscle group
ϕ =: pennation angle between muscle and tendon

## Appendix A: Calculating Muscle Length and Velocity Using a Rigid Tendon Model

The total length of the MT system $l^{\mathrm{MT}}$ is given by

(A1)
$$l^{\mathrm{MT}}=l^{M}\text{cos}\phi +l^{T}$$

Given the current joint angle q and the angular velocity $\dot{q}$, the current length, $l^{\mathrm{MT}}$ of the MT system can be calculated using trigonometric relations. As an example, consider the following simplified model (Fig. 13).

The total length and velocity of the MT complex can then be written as

(A2)
$$\begin{array}{c} l^{\mathrm{MT}}=\sqrt{l_{1}^{2}+l_{2}^{2}+2l_{1}l_{2}\text{cos}q}\\ v^{\mathrm{MT}}={\dot{l}}^{\mathrm{MT}}=\dot{q}(-l_{1}l_{2}\text{sin}q)/l^{\mathrm{MT}} \end{array}$$

where $l_{1},l_{2}$ are the projected lengths of the MT complex on the forearm and upper-arm. By using the fixed-height assumption to maintain a constant muscle volume

(A3)
$$l^{M}\text{sin}\phi =l_{0}^{M}\text{sin}\phi_{0}$$

the muscle length can then be found as

(A4)
$$l^{M}=\sqrt{{\left(l^{\mathrm{MT}}-l_{S}^{T}\right)}^{2}+{\left(l_{0}^{M}\text{sin}\phi_{0}\right)}^{2}\;}$$

using which the pennation angle ϕ can also be found. The velocity of the MT system can then be found as

(A5)
$$v^{M}=v^{\mathrm{MT}}\text{cos}\phi$$

## Appendix B: Hill-Type Muscle Models

For the length-dependent muscle force relations, this work uses the equations given in Ref. [48,49].

The active muscle force dependent on variation in length is given as

(B1)
fA,L(l~M)=9(l~M−0.4)2, l~M≤0.61−4(1−l~M)2, 0.6≤l~M≤1.4 9(l~M−1.6)2, l~M>1.4

(B2)
fP(l~M)=0, l~M≤1γ1(exp(γ2(l~M−1))−1), 1≤l~M≤1.4 (γ1γ2exp(0.4γ2))l~M+γ1((1−1.4γ2)exp(0.4γ2)−1), l~M>1.4

where $\gamma_{1}=0.075$ and $\gamma_{2}=6.6$ correspond to parameters in the passive muscle force model related to an adult human. The muscle force velocity relationship $f^{V}({\tilde{v}}^{M})$ is used directly from Ref. [50].

## Appendix C: Sensitivity Study of Weight Parameter β in Composite Loss Function

The scaling parameter β in the loss function in Eq. (14) was estimated as $\beta \propto \frac{\Delta t^{2}}{I}$, as described in Secs. 2.2.1 and 3. A sensitivity study performed in this appendix uses the verification problem in Sec. 3 to demonstrate the proper range of β for stable and accurate solution of optimization problem in Eq. (14). Six different parameters $\beta =10^{0},{10^{-1},10}^{-2},10^{-3},10^{-4},10^{-5}$ were chosen as shown in Fig. 14 and the results show the motion prediction of one representative training trial and the optimal MSK parameters identified for each β. The estimated $\beta \propto \frac{\Delta t^{2}}{I}=10^{-4}$ falls in the range of $\beta =[{10^{-5},10}^{-3}]$ where good optimization solutions are obtained.
